# Assembly and comparative analysis of the mitochondrial genome of *Pteroceltis tatarinowii* Maxim. (Cannabaceae), a tertiary relict plant endemic to China

**DOI:** 10.3389/fpls.2026.1925852

**Published:** 2026-10-21

**Authors:** Qiong Liu, Qi Jing, Beibei Cheng, Tong Liu, Lei Wang, Dan Liu

**Affiliations:** 1 Shandong Normal University, Jinan, China; 2 Shandong Petrochemical & Energy Group, Jinan, China; 3 Hebei Normal University of Science and Technology, Qinhuangdao, China; 4 Shandong Provincial Center of Forest and Grass Germplasm Resources, Jinan, China

**Keywords:** mitochondrial genome, *Pteroceltis tatarinowii* Maxim., repeated sequence analysis, sequence transfer, tertiary relict plant

## Abstract

**Introduction:**

*Pteroceltis tatarinowii* Maxim. (Cannabaceae) is a deciduous arbor endemic to China. As a Tertiary relict plant, it is listed as a rare and endangered species under national Class III key protection. Mitochondrial genomic data of *P. tatarinowii* can improve our knowledge of phylogenetic relationships within Cannabaceae, resolve the taxonomic placement and evolutionary divergence of the genus *Pteroceltis*, and supply molecular support for conservation and genetic breeding of this endangered tree.

**Methods:**

We sequenced and assembled the mitochondrial genome of *P. tatarinowii* using Illumina short reads and Oxford Nanopore long reads. Bioinformatic pipelines were applied for gene annotation and functional characterization. Repetitive sequences and chloroplast-derived transferred fragments were screened and systematically characterized.

**Results:**

The assembled mitochondrial genome of *P. tatarinowii* exhibited a typical multi-branched topology, with a total length of 509,000 bp and an overall GC content of 45.37%. A total of 182 simple sequence repeats, 12 tandem repeats and 117 dispersed repeats were detected, dominated by short repetitive motifs. Seven chloroplast homologous transferred fragments were annotated, spanning 660 bp and accounting for 0.13% of the mitochondrial genome. A maximum-likelihood phylogeny based on conserved mitochondrial coding sequences from 33 plant species firmly resolved *P. tatarinowii* in Rosales, Cannabaceae, consistent with the latest APG classification system.

**Discussion:**

This study reports the first complete mitochondrial genome of *P. tatarinowii*. The genome is characterized by abundant repetitive sequences and intergenomic sequence transfer from the chloroplast. These resources provide fundamental data for investigating mitochondrial genome evolution in Cannabaceae and lay a molecular foundation for future studies on endangerment mechanisms, genetic diversity assessment and conservation management of this relict endangered species.

## 1 Introduction

*Pteroceltis tatarinowii* Maxim., belongs to the genus *Pteroceltis* of the Cannabaceae family, is a deciduous arbor species endemic to China. It is considered a tertiary relict plant, and a rare and endangered plant under national Class III key protection (State Environmental Protection Administration and Institute of Botany, Chinese Academy of Sciences, 1987). This species is widely distributed in the temperate and subtropical regions of China. Among them, the subtropical areas between 25°N and 35°N have the most concentrated distribution. It grows sporadically in scattered forms in the valley, beside the stream, and on the limestone mountainous shrubland at an altitude of 100–1500 m (Fu et al., 2003). *P. tatarinowii* has an elegant shape and unique bark. It is an excellent tree species for landscaping and bonsai cultivation. The wood of *P. tatarinowii* is hard, dense, and highly resilient, making it a high-quality material for furniture, agricultural tools, and fine woodworking (Fang et al., 2002). In addition, the stem bark fibers of this tree species are of superior quality, making them the preferred primary raw material for producing Xuan paper. The seeds can be used for oil extraction, while the leaves can be used as animal feed. This species holds significant economic and cultural value (Fang et al., 2002). *P. tatarinowii* has strong adaptability, thriving in calcium-rich environments and sunlight, with some tolerance for shade, drought, and poor soil conditions. In its natural habitat, although the species has a high rate of fruiting, the natural seed reproduction ability is weak and affected by dormancy (Zhang et al., 2007). Additionally, the fragmentation of the habitats of wild populations and the deterioration of their living environment have severely restricted their natural regeneration, leading to a decline in some populations and posing a risk of a shrinking distribution area (Fang and Fu, 2007).

Currently, research on *P. tatarinowii*, both within the country and internationally, primarily focuses on seed morphological variation (Tang et al., 2018), community ecology (Zhang et al., 2011), genetic diversity (Zhu et al., 2016; Shu, 2015; Guo et al., 2024), breeding techniques (Wang, 2018), fiber structure (Gao et al., 2007), pests and diseases, and stress resistance (Li et al., 2016; Cao, 2005). Previous genomic and molecular studies have used chromosome-level genome assembly to analyze the evolutionary mechanisms of *P. tatarinowii* and the key genes involved in fiber biosynthesis (Qiao et al., 2025); developed and validated 20 new EST-SSR markers with high polymorphism and good stability for population genetics analysis (Zhang et al., 2020); and identified and published the first complete chloroplast genome, revealing its structural features and providing basic data for phylogenetic research (Zhang et al., 2019). Systematic studies on the mitochondrial genomes of tertiary relict plants, such as *P. tatarinowii*, are relatively scarce.

To clarify the structural characteristics of the *P. tatarinowii* mitochondrial genome, its phylogenetic placement within the Cannabaceae family, and the evolutionary patterns and molecular mechanisms of the *P. tatarinowii* mitochondrial genome, in this study, we sequenced, assembled, annotated, and systematically analyzed the mitochondrial genome of *P. tatarinowii.* We aimed to address the research gap concerning the mitochondrial genome of *P. tatarinowii* and provide key molecular evidence for the phylogenetic investigation of the *Pteroceltis* genus and Cannabaceae family. Additionally, this study establishes a theoretical basis for the conservation and sustainable utilization of the genetic resources of *P. tatarinowii*.

## 2 Materials and methods

### 2.1 Experimental materials and sequencing

The study utilized *P. tatarinowii* as the experimental material. Fresh young leaves were collected in December 2025 from a single *P. tatarinowii* tree (36.627436°N, 117.166417°E, 210 m a.s.l.) located at the Shandong Provincial Center of Forest and Grass Germplasm Resources. After collection, the samples were immediately frozen in liquid nitrogen and stored at −80 °C in an ultra-low-temperature freezer until use. Genomic DNA was extracted using a DNA extraction kit (Tiangen, China). DNA purity was determined with a NanoDrop 2000 spectrophotometer (Thermo Fisher Scientific, USA), and DNA concentration was quantified using a Qubit 4.0 fluorometer (Thermo Fisher Scientific, USA). DNA integrity was verified using agarose gel electrophoresis. Secondand third-generation sequencing libraries were constructed separately for combined sequencing (Liu et al., 2025). The second-generation short-read library was prepared by randomly shearing genomic DNA with a Covaris sonicator. After end repair, adapter ligation, and PCR amplification, the library was purified using 0.8× AMPure XP beads. Library quality was verified using an Agilent 2100 Bioanalyzer, followed by sequencing on the NovaSeq 6000 platform. The third-generation long-read library was prepared using the SQK-LSK114 kit according to the standard Oxford Nanopore Technologies (ONT) protocol, followed by DNA damage repair, end repair, dA-tailing, and adapter ligation, with no PCR amplification. The library was purified using 0.4× AMPure XP beads, and the fragment size distribution was verified using a Femto Pulse system. The final library was then loaded onto the PromethION platform for high-throughput sequencing. For the raw data obtained, quality control of the short-read and long-read datasets was performed using Fastp (v0.23.2) and NanoFilt (v2.8.0), respectively. Only reads with a quality score (Q) ≥ 19 were retained for subsequent genome assembly.

### 2.2 Genome assembly and annotation

After sequencing and quality filtering, reads corresponding to the *P. tatarinowii* mitochondrial genome were obtained. The assembly process utilized Flye (v2.9.2-b1786) (Kolmogorov et al., 2019) with default settings, relying on long-read data to produce a graphical assembly in GFA format. All contigs in FASTA format were used to create a local database using makeblastdb, and contigs belonging to the mitochondrial genome were identified and screened using the BLASTn program(parameters: -value 1e-5 -outfmt 6 -max_hsps 10 -word_size 7 -task BLASTn-short). The BLASTn search was performed against the publicly available mitochondrial genome sequences of closely related species deposited in the NCBI GenBank database, including the complete mitochondrial genomes of *Cannabis sativa* L.(GenBank: NC_029855.1) and *Humulus lupulus* L.(GenBank: NC_086845.1). The GFA file was visualized using Bandage (v0.8.1) (Wick et al., 2015), and mitochondrial contigs were further screened based on BLASTn results, ultimately yielding a draft mitochondrial genome of *P. tatarinowii*.

To obtain qualified reads for hybrid assembly, minimap2 (v2.26-r1175) (Li, 2018) was used to map both long-read and short-read data to the preliminary mitochondrial contigs, and the aligned mitochondrial reads were filtered and retained for subsequent assembly.Subsequently, based on the filtered long-read and short-read datasets, final mitochondrial genome hybrid assembly was performed using Unicycler (Wick et al., 2017) with default parameters. The assembled mitochondrial genome was visualized and verified again using Bandage (v0.8.1) (Wick et al., 2015). The entire mitochondrial genome was annotated using PMGA (http://www.1kmpg.cn/pmga/) (Li et al., 2025), tRNA genes were annotated using tRNAscan-SE (v.2.0.11) (Lowe and Eddy, 1997), and rRNA genes were annotated through BLASTn (v2.16.0) (Chen et al., 2015). Any errors in annotations were manually curated and corrected using Apollo (v1.11.8) (Lewis et al., 2002). Finally, the mitochondrial genome map was constructed and visualized with OGDRAW (Greiner et al., 2019). The complete assembly and annotation data have been deposited in the GenBank database of the National Center for Biotechnology Information (NCBI). The accession number for the *P. tatarinowii* mitochondrial genome is PZ239996 (https://www.ncbi.nlm.nih.gov/nuccore/PZ239996).

### 2.3 Codon usage preference and RNA editing site analysis

Protein-coding sequences in the genome were extracted using PhyloSuite (v1.1.16) (Zhang et al., 2020). Codon usage bias analysis of the mitochondrial protein-coding genes (PCGs) was performed using MEGA7 (v7.0) (Kumar et al., 2016), and the relative synonymous codon usage (RSCU) values were calculated. A codon with an RSCU value greater than 1 is defined as a preferred codon with a high usage frequency; an RSCU value less than 1 indicates low usage frequency; and an RSCU value of 1 suggests no obvious codon usage bias.

Deepred-mt software (Edera et al., 2021), which is based on a convolutional neural network (CNN) model, was used to predict C-to-U RNA editing sites within mitochondrial protein-coding genes (PCGs), and only the editing sites with a prediction probability greater than 0.9 were retained for subsequent analysis.

### 2.4 Analysis of repetitive sequences and homologous sequence transfer

Statistical analysis of microsatellite, tandem, and dispersed repeats in the mitochondrial genome was performed using TRF (v4.09) (https://tandem.bu.edu/trf/trf.unix.help.html) (Benson, 1999) and ROUSfinder (Wynn and Christensen, 2019). Simple sequence repeats (SSRs) were identified using MISA (v2.1) (https://webblast.ipk-gatersleben.de/misa/) (Beier et al., 2017), followed by manual curation. The analysis results were visualized using Excel (2021) software and the RCircos package (v0.69.9) (Zhang et al., 2013). The chloroplast genome was *de novo* assembled from second-generation short-read sequencing data using GetOrganelle (v1.7.7.0) (Jin et al., 2020); subsequent annotation and result correction of the chloroplast genome were accomplished using CPGAVAS2 (Shi et al., 2019), and CPGView software (Liu et al., 2023). To detect homologous sequence transfer events between mitochondrial and chloroplast genomes, whole-genome alignment was performed with BLASTn (v2.16.0) (Chen et al., 2015). The mitochondrial genome was set as the query and the chloroplast genome as the subject sequence, with parameters -evalue 1e-5 and -word_size 11. Alignments with sequence identity ≥70% and alignment length ≥100 bp were retained as candidate transferred homologous fragments, and the results were visualized using the RCircos package (v0.69.9) (Zhang et al., 2013).

### 2.5 Collinearity and phylogenetic analysis

Pairwise alignments of each mitochondrial genome were obtained using the BLASTn program with the following parameters:-value 1e-5, -word_size 9, -gapopen 5, -gapextend 2, -reward 2, -penalty -3. Sequences longer than 500 bp were retained as conserved collinear blocks. Based on the BLASTn results, a multiple synteny plot (MSP) was constructed using MCScanX (Wang et al., 2012).

The mitochondrial genomes of 32 species were retrieved from the GenBank database, together with the mitochondrial genome of *P. tatarinowii* (**Supplementary Table S1**). A phylogenetic tree was constructed based on the DNA sequences of 29 conserved mitochondrial protein-coding genes.The common genes were extracted using PhyloSuite software (v1.1.16) (Zhang et al., 2020), and multiple sequence alignment analysis was conducted using MAFFT software (v7.525) (Katoh and Standley, 2013). The phylogenetic tree was constructed using IQ-TREE software (v2.3.6) (Minh et al., 2020) based on the maximum likelihood (ML) method with the following parameters: --alrt 1000 -B 1000. The visualization of the phylogenetic tree results was accomplished using the ITOL software (v7) (Letunic and Bork, 2024).

## 3 Results

### 3.1 Structural characteristics of the mitochondrial genome of *P. tatarinowii*

Using software visualization, a draft map of the *P. tatarinowii* mitochondrial genome was created from long-read data (**Figure 1**). This genome comprised six contigs (**Supplementary Table S2**), which were linked together forming a multibranched configuration within the assembly graph. By extracting and simplifying one possible sequence path as contig1-contig4-contig5-contig2-contig6-contig3, a typical multi-branched topology contig was obtained (**Figure 2**). This linearized contig path is only for visualization and does not represent the native molecular conformation. The *P. tatarinowii* mitochondrial genome exhibits a typical multi-branched topology, with a total length of 509,000 bp and a GC content of 45.37%.

**Figure 1:**
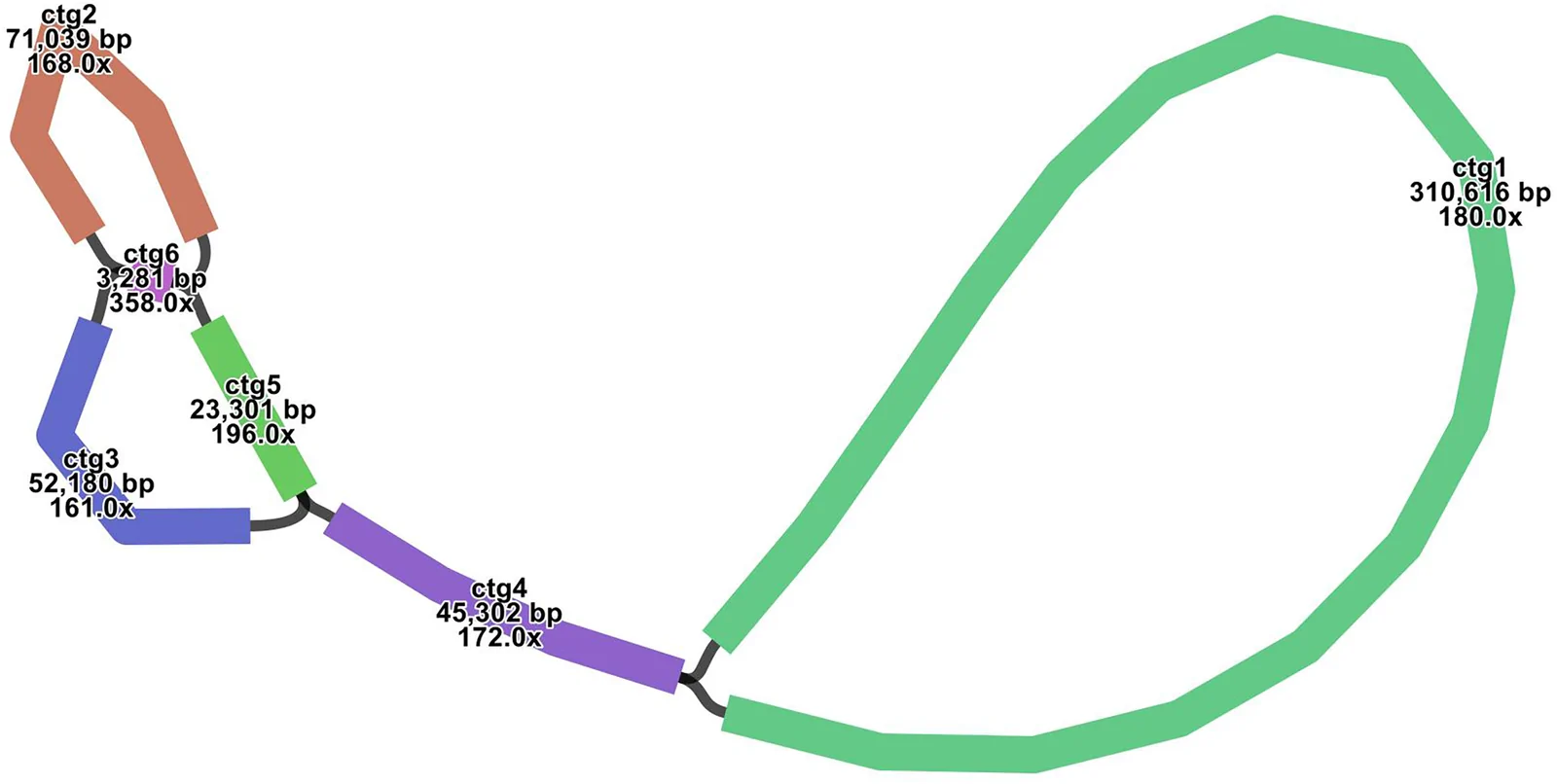
Draft mitochondrial genome of *P. tatarinowii.*.

**Figure 2:**
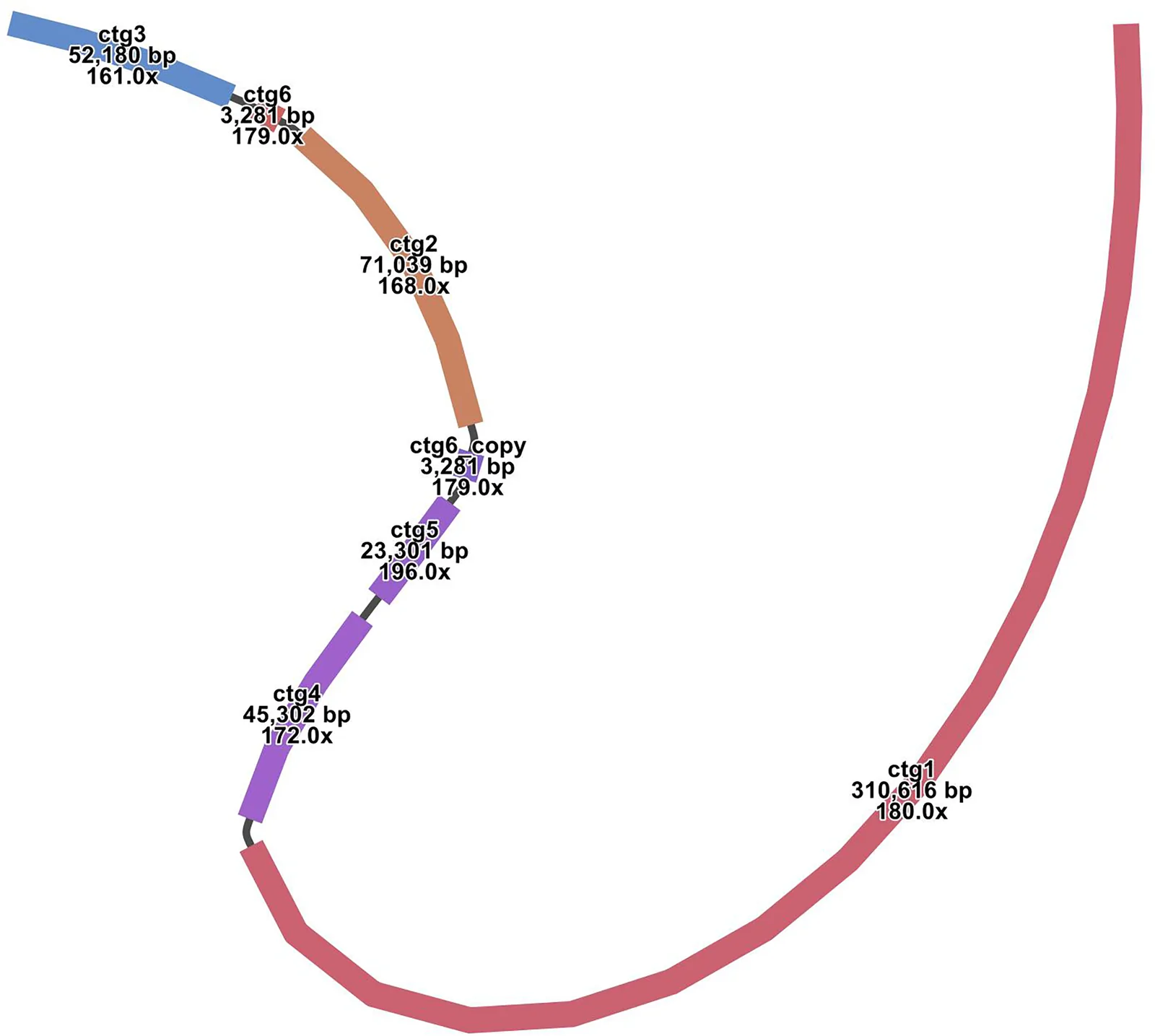
Schematic of contig connections for the *P. tatarinowii* mitochondrial genome.

In total, 56 genes were annotated in the multibranched mitochondrial genome of *P. tatarinowii*, including 16 tRNA genes (2 present in multiple copies); 3 rRNA genes; and 37 PCGs (1 present in multiple copies). Among these, there are 24 unique mitochondrial core genes and 13 non-core genes (**Figure 3**; **Supplementary Table S3**). Based on their functions, these genes can be divided into 10 categories: ATP synthase (six genes: atp1, atp4, atp6 [×2], atp8, and atp9); NADH dehydrogenase (nine genes: nad1,nad 2, nad3, nad4, nad4L,nad 5, nad6, nad7, nad9); cytochrome C biosynthesis (four genes: ccmB, ccmC, ccmFC, ccmFN); Cytochrome C oxidase (three genes: cox1, cox2, cox3); Membrane transport protein (one gene: mttB); Maturase (one gene: matR) and Cytochrome b (one gene: cob). The non-core genes included, large ribosomal subunit (three genes: rpl5, rpl10, rpl16), small ribosomal subunit (eight genes: rps3, rps4, rps7, rps10, rps12, rps13, rps14, rps19), and succinate dehydrogenase (two genes: sdh3, sdh4).

**Figure 3:**
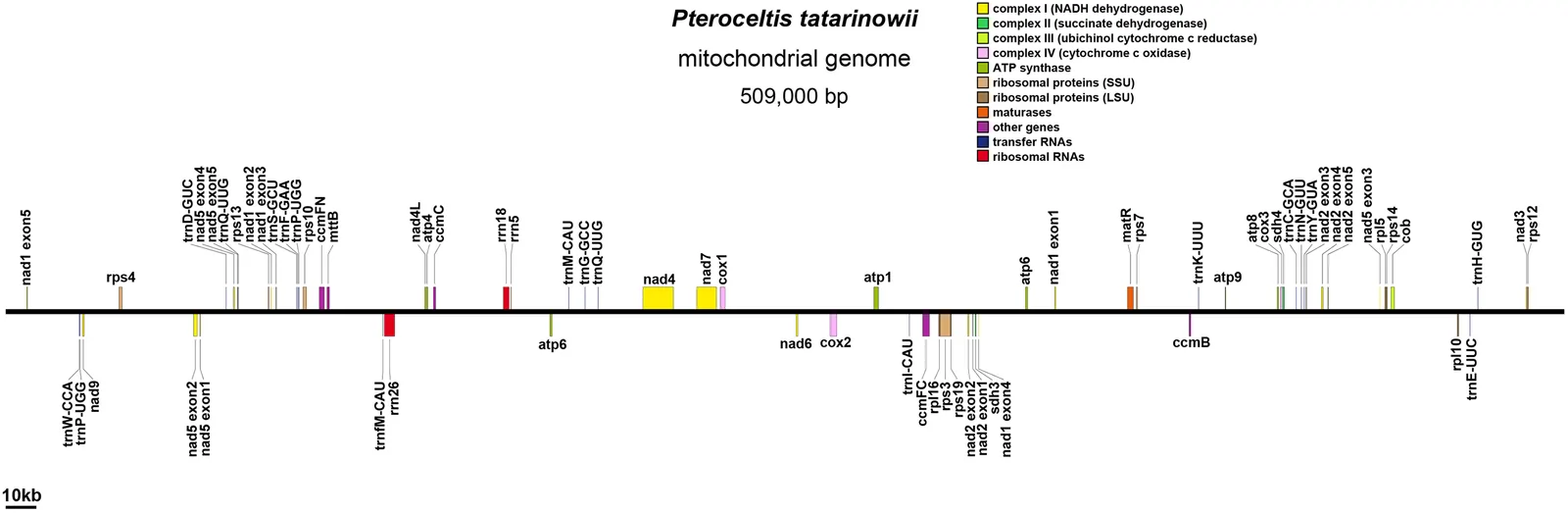
Linear map of the mitochondrial genome of *P. tatarinowii*.

### 3.2 Codon usage and RNA editing site analysis

The codon usage bias for the 37 protein-coding genes in the *P. tatarinowii* mitochondrial genome was analyzed (**Figure 4**; **Supplementary Table S4**). Amino acids with only two synonymous codons, including asparagine (Asn), aspartic acid (Asp), cysteine (Cys), glutamine (Gln), glutamic acid (Glu), histidine (His), lysine (Lys), phenylalanine (Phe), and tyrosine (Tyr), demonstrated a clear codon bias, with the relative synonymous codon usage (RSCU) values of their preferred codons greater than 1. The RSCU values of the start codon AUG (encoding Met) and the UGG codon for tryptophan (Trp) were both 1, suggesting no codon usage bias. Among all amino acids, alanine (Ala) for the GCU codon and His for the CAU codon exhibited the strongest codon preference, with RSCU values of 1.58 and 1.51, respectively. In contrast, the CAC and UAC codons for His and Tyr, respectively, exhibited the least preference, with RSCU values of 0.49 and 0.47, respectively.

**Figure 4:**
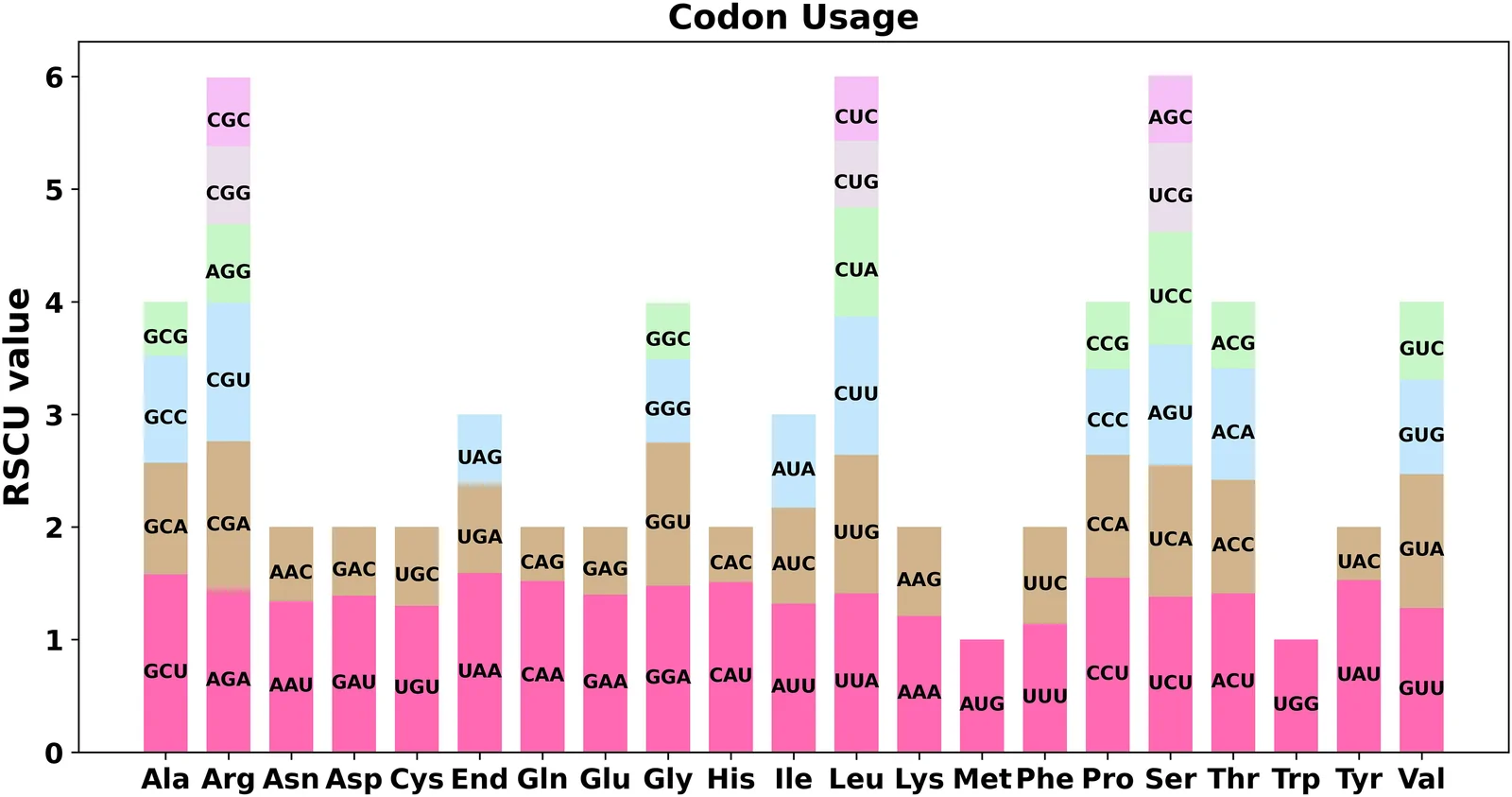
Codon usage preference analysis in the *P. tatarinowii* mitochondrial genome.

RNA editing, a vital post-transcriptional modification process in eukaryotes, alters the nucleotide sequences of mRNAs at specific editing sites, thereby influencing protein structure and function. This process is vital for maintaining the stability of the respiratory chain in plant mitochondria. As shown in **Figure 5**, a total of 455 potential RNA editing sites were identified across the 37 mitochondrial protein-coding genes of *P. tatarinowii*, demonstrating a high degree of base specificity, all of which were edited from base C to U. This pattern aligns with the typical mitochondrial RNA editing observed in most angiosperms. Among the mitochondrial genes, the nad4 gene harbored the highest number of potential editing sites (47) and exhibited the highest editing frequency, indicating that it is the key gene for RNA editing in *P. tatarinowii* mitochondria. This was followed by the nad7 gene (35 sites each), ccmC (34 sites each), and nad2 (33 sites each). The rpl10 and sdh4 had the fewest editing sites, with only one site identified in each.

**Figure 5:**
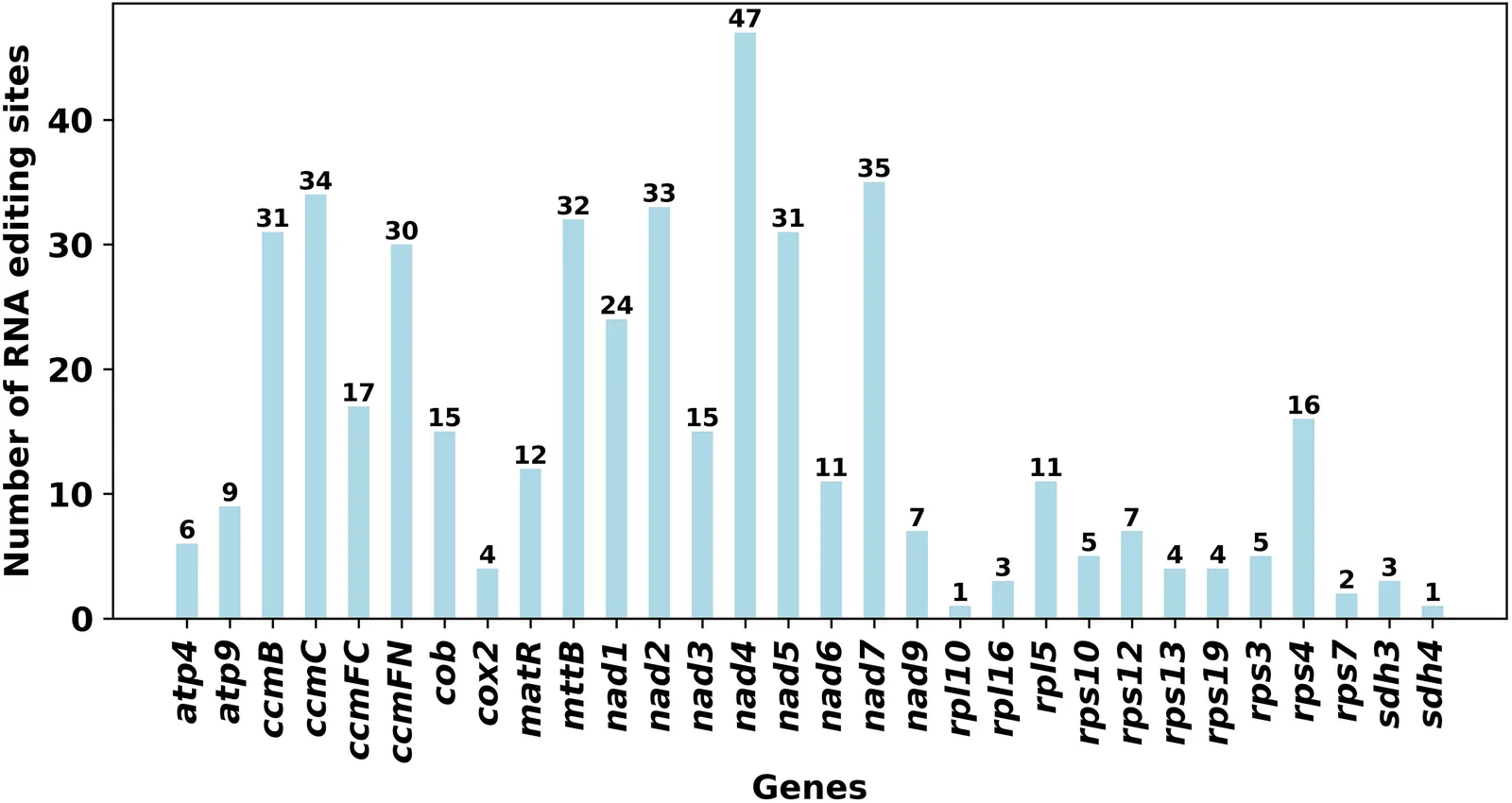
Distribution and number of RNA editing sites in *P. tatarinowii* mitochondrial genome.

### 3.3 Analysis of repetitive and homologous transferred sequences

Simple sequence repeats (SSR), also known as microsatellite DNA, are composed of 1–6 nucleotide bases as the basic repeating unit and are repeated multiple times in series. They have the characteristics of wide distribution, rich polymorphism, and codominant inheritance (Zhu et al., 2019). A total of 182 eligible SSRs were detected in the mitochondrial genome of *P. tatarinowii*, including 50 monomers, 41 dimers, 16 trimers, 66 tetramers, 7 pentamers, and 2 hexamers. As shown in **Figure 6**, the SSRs within the mitochondrial genome of *P. tatarinowii* exhibited distinct type specificity and base preference. Specifically, 66 tetramers were identified, accounting for 36% of all SSR types; 91 monomers and dimers were found, constituting 50% of the SSR types; while only 9 pentamers and hexamers were detected, representing less than 5% of the SSR types. Among the monomers, thymine (T) was the most prevalent accounting for 62% (31 occurrences), while adenine (A) monomer repeats accounted for 28% (14 occurrences), ranking second only to thymine (T). Additionally, there were two guanine (G) and three cytosine (C) monomer repeats.

**Figure 6:**
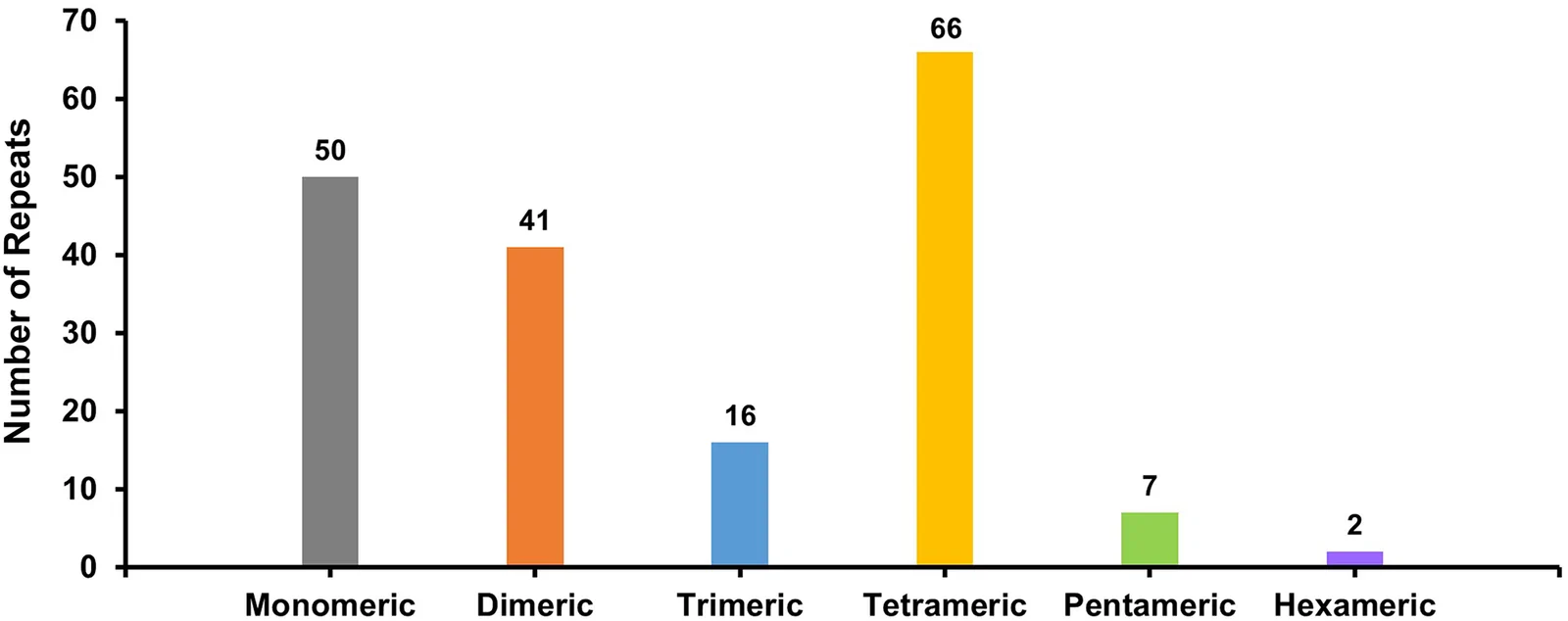
Proportion of various types of simple sequence repeats in *P. tatarinowii* mitochondrial genome.

A tandem repeat sequence (TRS), also known as satellite DNA, is a DNA sequence formed by consecutive head-to-tail repetitions of DNA motifs ranging in length from 1 to 200 bp. Based on the length of the repeat units, TRS can be classified into satellite DNA, minisatellites, and microsatellites (SSRs), which are characterized by high polymorphism and codominant inheritance (Zhu et al., 2019). Collectively, 12 relatively long tandem repeat sequences were identified in the mitochondrial genome of *P. tatarinowii*, with sequence identities greater than 81% and lengths ranging from 13 to 39 bp. We identified 117 pairs of dispersed repeat sequences with lengths ≥ 30 bp, including 54 pairs of palindromic repeats (the longest being 756 bp) and 63 pairs of forward repeats (the longest being 3281 bp) (**Supplementary Table S5**).

Sequence similarity analysis (**Figures 7**, **8**; **Supplementary Table S6**) identified seven chloroplast-derived homologous fragments, with a total length of 660 bp, in the mitochondrial genome of *P. tatarinowii*, accounting for 0.13% of the total mitochondrial genome length. MTPT3 and MTPT7 were the longest fragments, each measuring 148 bp. Annotation of these homologous sequences revealed four complete genes distributed across the seven fragments, all of which were tRNA genes (trnD-GUC, trnH-GUG, trnM-CAU, trnN-GUU).

**Figure 7:**
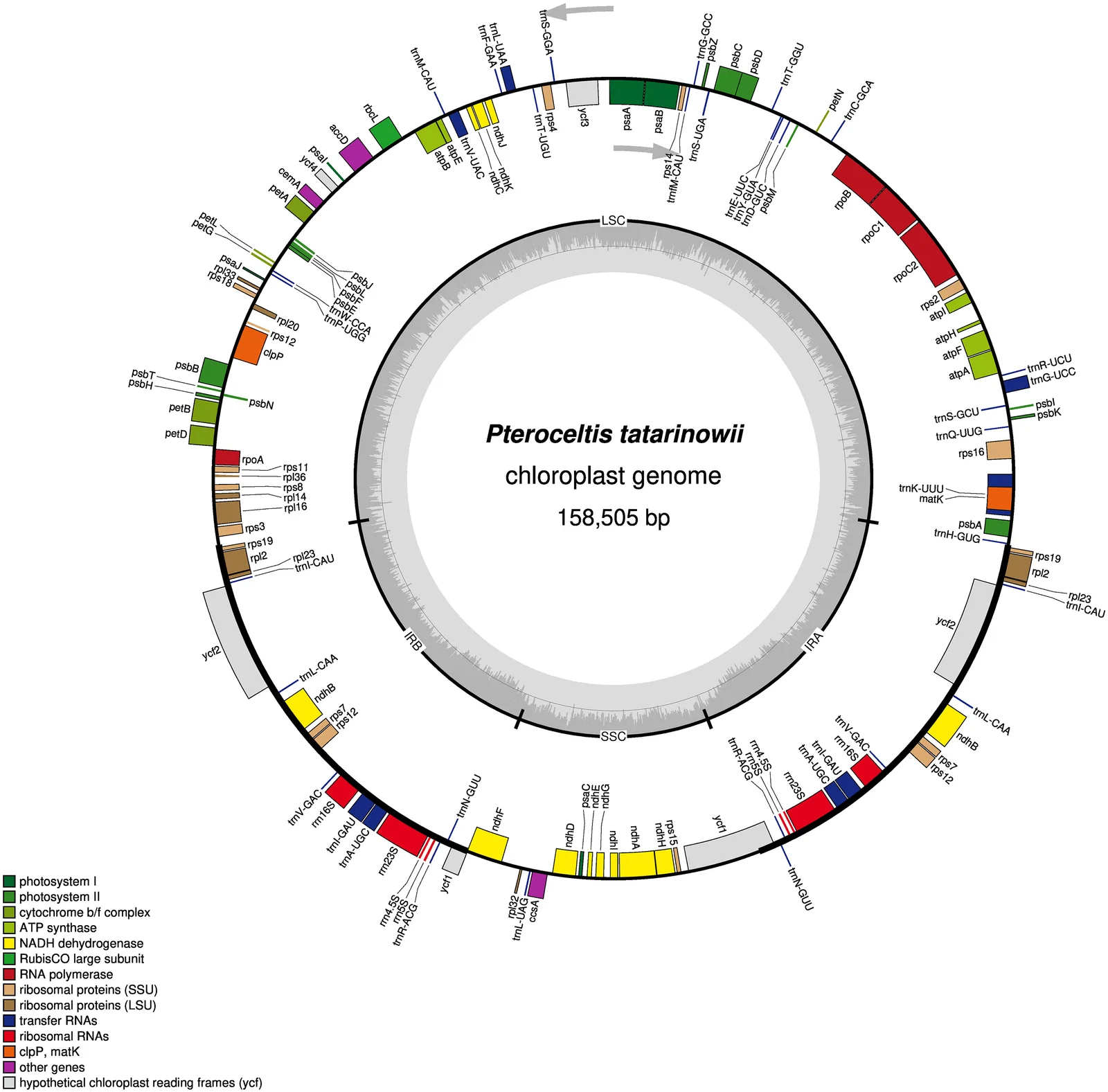
Circular map of the chloroplast genome of *P. tatarinowii*.

**Figure 8:**
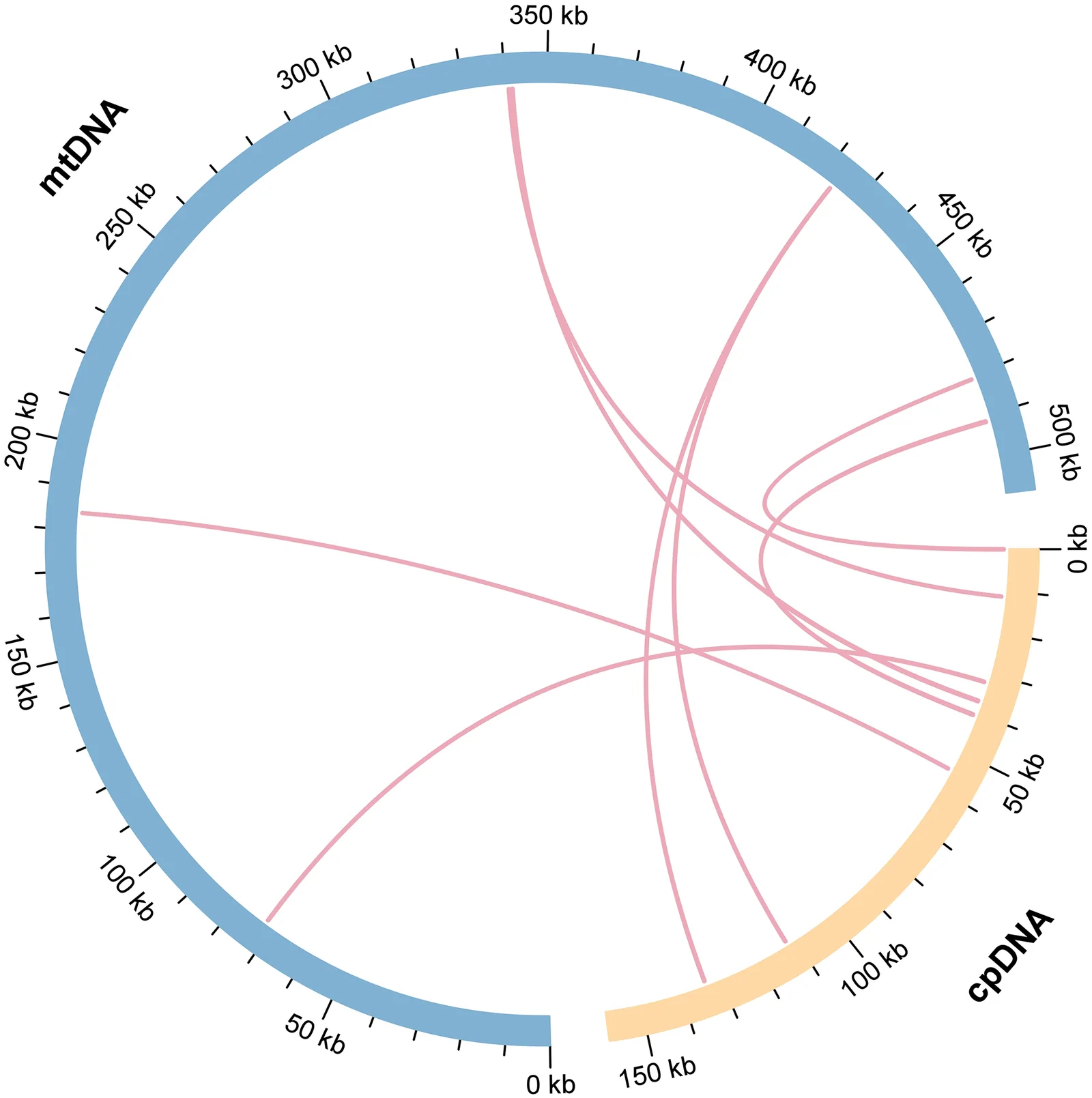
Analysis of homologous sequence transfer in *P. tatarinowii* mitochondrial genome.

### 3.4 Synteny and phylogenetic analysis

We conducted a collinearity comparison of mitochondrial genomes among three closely related species in the family Cannabaceae (**Figure 9**). The mitochondrial genomes of the three species exhibited high homology and a close evolutionary genetic relationship, consistent with the conservative nature of mitochondrial genomes. Homologous fragments frequently exhibited reversed orientations in *P. tatarinowii* when compared to *C. sativa* and *H. lupulus* (d.start>d.end). More than 60% of collinear blocks represented inversion events, indicating that the mtDNA of *P. tatarinowii* has experienced large-scale and high-frequency fragment inversions. A total of 82 valid collinear fragments were detected between *P. tatarinowii* and *H. lupulus*, and 70 collinear fragments were identified in the mitochondrial genomes of *P. tatarinowii* and *C. sativa*. The longest sequence match was merely 9141 bp; no long continuous collinear gene clusters were retained. Although individual homologous genes remain highly conserved at the sequence level, these collinear fragments have undergone extensive positional reshuffling. The distribution positions of homologous blocks on *P. tatarinowii* mtDNA were entirely disordered compared to the corresponding regions in *C. sativa* and *H. lupulus*, showing an interwoven, displaying an interwoven, discontinuous, and scattered distribution pattern. This observation demonstrates that, despite high conservation of individual gene sequences, the mitogenome of *P. tatarinowii* has suffered substantial structural rearrangements during evolution, leading to poor conservation in fragment arrangement and genomic architecture.

**Figure 9:**
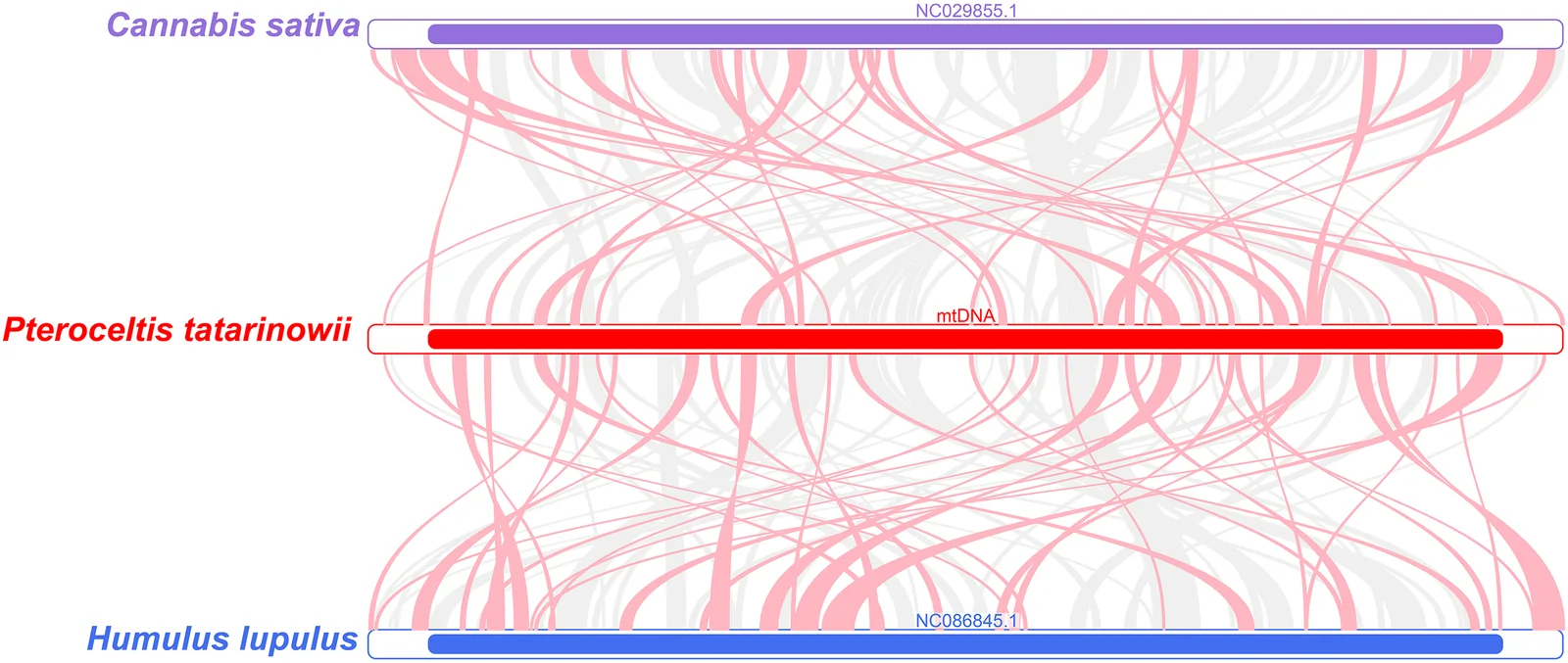
Homology analysis of mitochondrial genomes.

The mitochondrial genomes of 32 species were downloaded from GenBank in this study, together with the mitochondrial genome of *P. tatarinowii*. The resulting 33 species belong to two orders, namely Rosales and Fabales (**Supplementary Table S1**). By comparing and examining the variations, such as deletion, insertion, duplication, and translocation of DNA sequences, this study indirectly clarifies the complex evolutionary rules and processes of plant mitochondrial genomes. To further explore the evolutionary patterns, a phylogenetic tree was constructed using the mitochondrial genome sequences from 33 species that had been sequenced previously focusing on the DNA sequences of 29 conserved protein-coding genes (PCGs) (**Figure 10**). The phylogenetic topology derived from mitochondrial DNA corresponds with the most recent classification by the Angiosperm Phylogeny Group (APG). Species from the Rosaceae and Fabaceae families formed monophyletic groups with strong support, and the two mitochondrial genomes from the Fabaceae family were classified as outgroups. These results confirm that *P. tatarinowii* belongs to the Rosales order and the Cannabaceae family.

**Figure 10:**
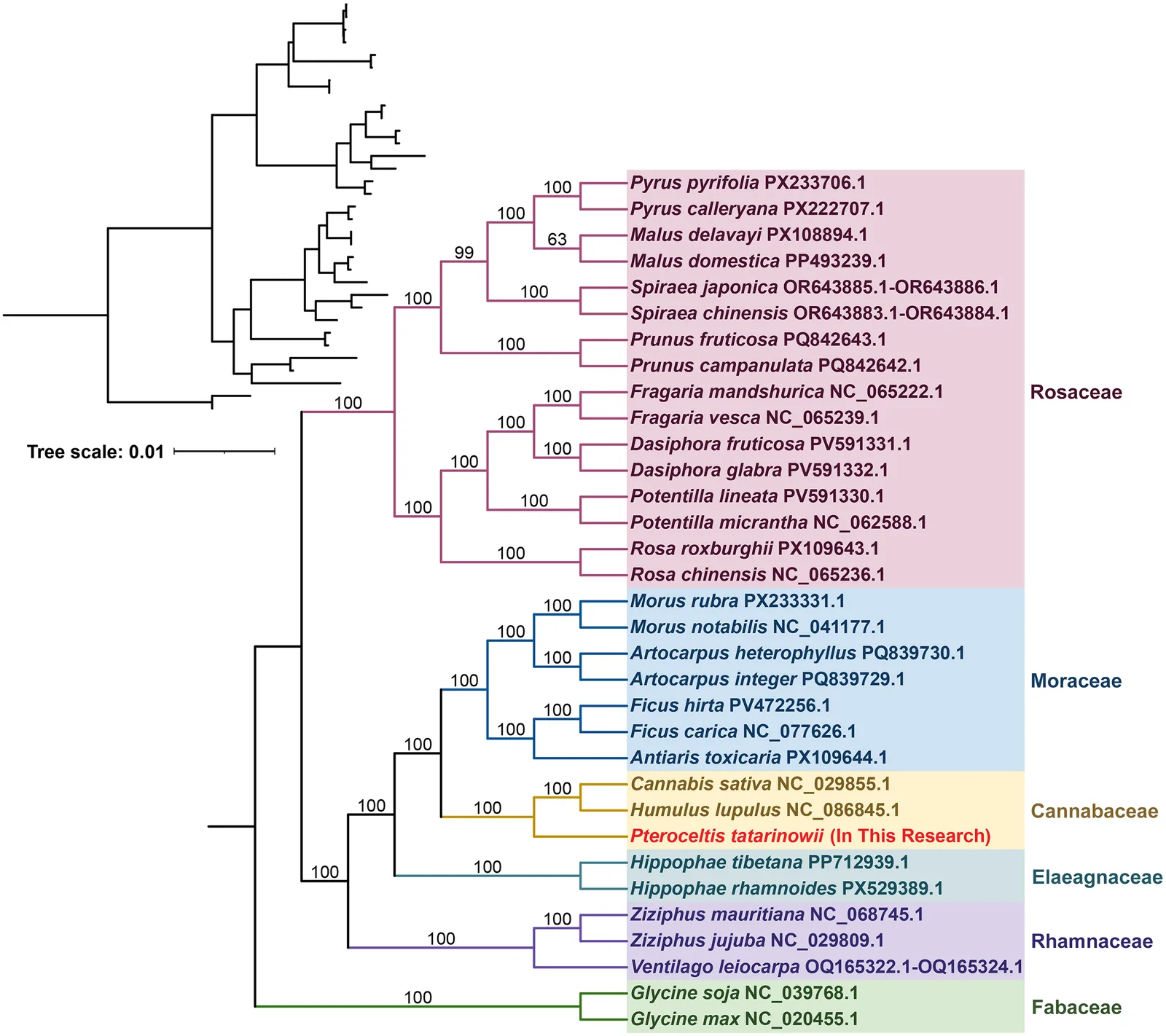
Phylogenetic analysis of *P. tatarinowii* and related species based on conserved mitochondrial genes.

The phylogenetic tree constructed in this study showed that three species within the Cannabaceae family form a monophyletic branch with strong support values. This finding reinforces the classification of Cannabaceae as a distinct family-level taxonomic unit, clearly separating it from closely related groups, such as Moraceae and Rosaceae. Within the Cannabaceae family, the topological structure displays a clear hierarchical differentiation: the *Pteroceltis* genus is located at the base of the family and is the earliest differentiated branch, whereas the *Cannabis* and *Humulus* genera form a secondary monophyletic branch and are sister genera with a closer genetic relationship. The branch lengths suggest that the divergence times between *P. tatarinowii* and *C. sativa*, as well as *H. lupulus*, occurred earlier than those between *Cannabis* and *Humulus*, implying that *P. tatarinowii* retains more ancestral traits and is a key group for analyzing the origin and evolution of the Cannabaceae family. At the family level of Rosales, Cannabaceae and Moraceae are sister groups. The two families first cluster into a single clade and then diverge sequentially with Rosaceae, Rhamnaceae, Elaeagnaceae, and other families. These taxa collectively form the core monophyletic group of Rosales, which is consistent with the latest phylogenetic framework based on nuclear genomes and multiple gene fragments. The findings of this study provide robust topological evidence for the genomic evolution of *P. tatarinowii*, the reclassification of the Cannabaceae family, and the phylogenetic framework of the Rosales order, which is significant for enhancing the phylogenetic and taxonomic system of Rosales.

## 4 Discussion

Mitochondrial genomes of higher plants exhibit complex structural variations, while chloroplast genomes are mostly circular double‐stranded DNA molecules, ranging in length from 120 to 160 kb, with highly conserved structure and gene composition (Palmer, 1985). Plant mitochondrial genomes are the most structurally complex among eukaryotic genomes, with a size ranging from 200 to 2500 kb. They often coexist with multiple isoforms in a primary circular form, with abundant repetitive sequences and recombination hotspots, with frequent structural rearrangements, numerous introns, and transfer fragments of chloroplast DNA. However, mitochondrial genomes exhibit a slow evolutionary rate but a rapid rate of structural variation (Newton, 1988).

*P. tatarinowii* is a deciduous tree of the Cannabaceae family. There is limited research on the mitochondrial genomes of Cannabaceae, and their genome sizes vary significantly. In 2023, Liu et al. performed hybrid sequencing using Oxford Nanopore long reads and Illumina short reads, and revealed that the total length of the *C. sativa* mitochondrial genome is 415,837 bp with a branched multimeric dynamic structure, among which the circular conformation as the predominant form (Liu et al., 2023). Christenhusz et al (Christenhusz et al., 2025). sequenced *H. lupulus* in 2025 using a combination of PacBio HiFi long reads, Illumina NovaSeq 6000 short reads, and Hi-C chromatin conformation capture. The assembled mitochondrial genome measured 400,020 bp with a typical circular structure. In contrast, the total length of the mitochondrial genome of *P. tatarinowii* assembled with annotations is 509,000 bp, which is larger than the mitochondrial genomes of the two species reported above. The structure of *P. tatarinowii* genome differs from that of *C. sativa* and *H. lupulus*, presenting a typical multi-branched topology.

RNA editing involves base conversion, insertion, or deletion in the coding regions of RNA after transcription, and is an important post-transcriptional regulatory mechanism that indirectly repairs DNA mutations and regulates gene expression at the RNA level. In a study of RNA editing events in three species in the Cannabaceae family, a total of 455 potential RNA editing sites were identified on the mitochondrial PCGs of *P. tatarinowii* in our present analysis. These included 432 non-synonymous editing sites and 23 synonymous editing sites; all of which were cytidine-to-uridine (C-to-U) base conversion, showing a marked preference for certain genes. The nad4 gene had the highest number of editing events (47, 10.3%), followed by nad7 (35, 7.7%), ccmC (34, 7.5%). Mitochondrial-related genes (nad family) and ccm family genes were the main editing targets. The distribution of editing events across codons was highly uneven, with the second base of the codon having the strongest function (261, 57.4%), followed by the first base (173, 38.0%), and the third base (21, 4.6%). For reference, published data reported a total of 28 RNA editing sites were detected in 38 mitochondrial PCGs of *C. sativa*, of which 14 sites were located in the protein-coding regions and 14 in the intergenic regions. Experimental verification confirmed eight editing sites in the coding regions, including four synonymous editing sites and four non-synonymous editing sites. The dominant editing type was C-to-U conversion, with a few guanine-to-adenine (G-to-A) events; all detected edits were transition-type modifications with no transversions. The number of editing sites was lower than that in model plants, and these sites showed clear tissue specificity, with more editing events detected in roots and stems than in leaves (Liu et al., 2023). Likewise, total of 480 RNA editing sites (441 non-synonymous edits and 39 synonymous edits) were detected in 36 PCGs of the *H. lupulus* mitochondrial genome. Among them, C-to-U editing (482 sites, 99.2%) was the dominant type, and there were no abnormal editing types (Christenhusz et al., 2025).It should be noted that these two datasets were generated by independent analytical pipelines instead of our workflow for P. tatarinowii, so direct quantitative comparisons should be interpreted with caution.

In contrast, all three species conform to the universal rule of mitochondrial RNA editing in angiosperms, in which the conversion from C to U predominates. This demonstrates the high conservation of the mitochondrial editing mechanism in terrestrial plants. However, there were significant differences in editing quantity, site preference, secondary editing types, and tissue specificity. The number of RNA editing sites in *P. tatarinowii* and *H. lupulus* was much higher than that in *C. sativa*, indicating substantial divergence in the intensity of mitochondrial RNA editing among different genera within Cannabaceae. The genus *Cannabis* may have experienced an evolutionary event resulting in massive loss of editing sites.

Plant mitochondrial genomes generally contain various types of repetitive sequences, including tandem, simple, inverted, and dispersed repeats (Gualberto and Newton, 2017). These repetitive sequences mediate homologous recombination. This phenomenon is widespread and promotes the formation of various genome conformations, playing a crucial role in structural variations and the evolution of plant mitochondrial genomes (Zhong et al., 2022). A study was performed to compare the repetitive sequences in the mitochondrial genomes of three plants from the Cannabaceae family: *P. tatarinowii* contained 182 SSRs (**Supplementary Table S7**), 12 tandem repeats, and 117 scattered repeats; with only one long scattered repeat (with a length of 3281 bp) exceeding 1 kb; the repetitive sequences made up 8.63% of the genome, with short fragment repeats being predominant. *C. sativa* had 107 SSRs (60.92% of which are single-nucleotide units), 20 tandem repeat sequences (with lengths ranging from 13 bp to 39 bp), and 38 dispersed repeat sequences (the longest being 486 bp). Through PCR verification, it was confirmed that 15 pairs of these repeat sequences underwent recombination events, highlighting that repeat sequences are crucial for maintaining the structural polymorphism of the mitochondrial genome (Liu et al., 2023). *H. lupulus* had 326 SSRs with lengths ranging from 20 to 100 bp and showed the highest degree of scattered repeats, suggesting more frequent repeat expansion events in its mitochondrial DNA. *H. lupulus* mitochondrial DNA has 17 families of tandem repeats, mostly of medium-to-short-cycle; and 412 pairs of scattered repeats, including two long fragments over 1 kb in length, with the longest one reaching 3845 bp (Christenhusz et al., 2025). The comparison indicates that all three species conform to the core rule that the length of repetitive sequences is positively correlated with the frequency of recombination, and large-scale repetitive sequences are more likely to drive genomic structural variations. However, there are significant differences in repeat quantity, length distribution, proportion, and recombination activity, which reflects species-specificity in the dynamic evolution of mitochondrial genomes within Cannabaceae.

During mitochondrial evolution, partial chloroplast fragments migrate into the mitochondrial genome, and the length and sequence similarity of the migrated fragments vary across species (Stern and Palmer, 1984). The phenomenon of sequence migration from the chloroplast genome has been identified in the mitochondrial genomes of three members of the family Cannabaceae. *P. tatarinowii* contains 7 fragments (with a total length of 660 bp, accounting for 0.13% of the mitochondrial genome), the longest fragments are MTPT3 and MTPT7 (148 bp); it contains 4 complete genes (trnH-GUG, trnN-GUU, trnD-GUC, trnM-CAU), and it is hypothesized that tRNA genes may serve as migration hotspots; sequence similarity ranges from 91.89% to 100%, and the overall homology is relatively high. *C. sativa* contains 19 segments (with a total length of 5315 bp, accounting for 1.27%), including 7 complete tRNA genes and 9 plastid gene segments. Additionally, a large number of frequent and extensive DNA migrations among the mitochondrial, nuclear, and plastid genomes have been identified, playing a crucial role in genomic evolution (Liu et al., 2023). *H. lupulus* contains 18 fragments (with a total length of 9933 bp, accounting for 2.48%), the longest fragment is 1124 bp, including trnH-GUG and psbA; it also contained 6 complete chloroplast tRNAs and 8 plastid protein-coding gene fragments (Christenhusz et al., 2025).

Upon analyzing and comparing the three species, it was found that all exhibited a preference for tRNA gene transfer, with high sequence homology. This is consistent with the previously reported characteristic of plant MTPTs, in which tRNA genes are prone to migration. However, the migration and evolution strategies of MTPTs in the three species differed: *P. tatarinowii* had the fewest MTPTs and the most conservative genomic evolution; *C. sativa* had frequent genomic interactions; and *H. lupulus* had the largest scale of transfer and the longest fragments. These differences highlight the diverse evolutionary patterns of mitochondrial genome within the Cannabaceae family, driven by differences in life history, mitochondrial recombination activity, and selective pressures across different lineages.

Collinearity analysis was used to decipher the linear arrangement patterns of homologous sequences and genes and to investigate the structural characteristics and evolutionary dynamics of the mitochondrial genomes of three plant species in the Cannabaceae family. From a sequence consistency perspective, the average identity of collinear fragments between *P. tatarinowii* and *H. lupulus* is slightly higher (approximately 96.7%) than that between *P. tatarinowii* and *C. sativa* (approximately 96.1%). Moreover, long continuous matched fragments (up to 9141 bp) are found between *P. tatarinowii* and *H. lupulus*, suggesting a closer structural relationship between the mitochondrial genomes of these two species. The identity of collinear fragments among the three species generally exceeds 94%, with some short fragments even exceeding 99%, indicating that the core coding regions and functional sequences are highly conserved, which is consistent with the common characteristics of angiosperms. Regarding structural collinearity, the mitochondrial genomes of the three species exhibit a large number of homologous blocks, accompanied by local inversions, fragment rearrangements, and insertions/deletions. The collinear blocks of the three species are not arranged in a completely linear manner but are interlaced across the genomes, with some blocks showing reversed orientations (such as d.start > d.end), reflecting small-scale structural rearrangements following species differentiation. The alignment results show a generally low number of gaps (0–84) and mismatches (2–126), which further supports the stable structure and limited rearrangement events of mitochondrial genomes. The findings indicate that the mitochondrial genomes of the three Cannabaceae species exhibit good overall collinearity and high sequence conservation, with only local rearrangements and minor variations. This observation aligns with the general principle of slow evolution and structural conservation of mitochondrial genomes in angiosperms (Palmer et al., 2000), and also provides crucial evidence at the mitochondrial level for the phylogeny and genomic evolution of the Cannabaceae family.

The phylogenetic analysis based on mitochondrial protein-coding genes provides molecular evidence for the classification of plants in the Cannabaceae family. In this study, 29 conserved PCGs were used to construct the phylogenetic tree, including core gene families such as atp, cox, and nad. The results showed that the topological structure was consistent with the APG classification system, supporting the monophyly of the Cannabaceae family. The species is distinctly classified within the Cannabaceae family of the order Rosales, and forms a sister clade with *C. sativa* and *H. lupulus*, all members of the Cannabaceae family. Although the phylogenetic tree topologies derived from different functional genomes (such as complex I, III, etc.) are slightly different, but the family-level classification remains consistent. This study not only confirms traditional classification methods, but also demonstrates the usefulness of conserved mitochondrial PCGs in high-level classification. Gene transfer events can be traced through branching patterns, and variations in the selective pressure of functional genes may fine-tune local topologies, providing multi-dimensional molecular evidence for exploring the dynamic evolution of mitochondrial genomes.

## 5 Conclusions

In this study, the mitochondrial genome of *P. tatarinowii* was explored. After assembly and annotation, the results revealed that the mitochondrial genome of *P. tatarinowii* is a typical multi-branched topology with a total length of 509,000 bp and a GC content of 45.37%. A total of 56 genes were annotated, including 16 tRNA genes, 3 rRNA genes, and 37 PCGs. Moreover, 455 RNA editing sites were identified within the 37 PCGs. The genome of *P. tatarinowii* contained 182 SSRs, 12 tandem repeats, and 117 pairs of scattered repeats, primarily consisting of short fragment repeats. Furthermore, seven chloroplast homologous fragments (with a total length of 660 bp) were identified, accounting for 0.13% of the total length of the mitochondrial genome. The phylogenetic tree constructed using the mitochondrial genomes of 33 species has clarified the phylogenetic relationships of Cannabaceae and enabled a more precise resolution of the phylogenetic position of *Pteroceltis* within Cannabaceae. This study provides a molecular basis for the conservation and genetic breeding of *P. tatarinowii*.

## Data Availability

The complete assembly and annotation data have been uploaded to the National Center for Biotechnology Information GenBank database(https://www.ncbi.nlm.nih.gov/). The accession number is PZ239996.
